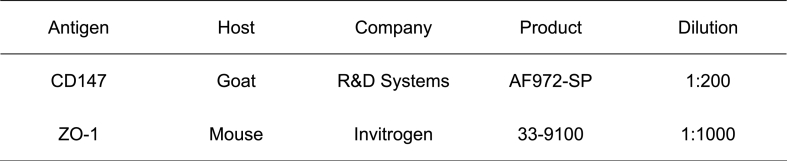# Investigation of the effectiveness of gelatin hydrolysate in human iPS-RPE cell suspension transplantation

**DOI:** 10.1016/j.reth.2023.12.016

**Published:** 2024-01-11

**Authors:** Shohei Kitahata, Michiko Mandai, Hinako Ichikawa, Yuji Tanaka, Toshika Senba, Keisuke Kajita, Sunao Sugita, Kazuaki Kadonosono, Masayo Takahashi

**Affiliations:** a Department of Ophthalmology and Micro-technology, Yokohama City University, 4-57 Urafunecho, Minami-ku, Yokohama, Japan; b Kobe City Eye Hospital, 2-1-8 Minatojima-minamimachi, Chuo-ku, Kobe, Japan; c Tokyo Metropolitan Geriatric Hospital and Institute of Gerontology, 35-2 Sakae-cho, Itabashi-ku, Tokyo, Japan; d Vision Care Inc., Kobe Eye Center 5F, 2-1-8 Minatojima-minamimachi, Chuo-ku, Kobe, Japan; e Department of Ophthalmology, Tokushima University, 2-24 Shinkura-cho, Tokushima, Japan

**Keywords:** Retinal pigment epithelium, Suspension transplantation, Induced pluripotent stem cells, iPS cell therapy, Gelatin, Regenerative medicine

## Abstract

**Introduction:**

The retinal pigment epithelium (RPE) plays essential roles in maintaining retinal functions as well as choroidal capillaries and can lead to visual disorders if dysfunctional. Transplantation of human-induced pluripotent stem cell-derived RPE (hiPSC-RPE) is a promising therapy for such RPE impaired conditions including age-related macular degeneration. The challenge with cell suspension transplantation is targeted delivery of graft cells and undesired cell reflux. Gelatin hydrolysate, a soluble variant with specific molecular weight distribution, is examined in this study for its potential use in hiPSC-RPE suspension transplantation, particularly in reducing cell reflux and enhancing RPE engraftment.

**Methods:**

A retinal bleb model was created using polydimethylsiloxane (PDMS) soft lithography to quantify cellular reflux. We examined the effects of gelatin hydrolysate on the hiPSC-RPE of various aspects of cell behavior and performance such as cell viability, hypoxia reaction, morphology, induction of inflammation and immune responses.

**Results:**

Gelatin hydrolysate at 5 % concentration effectively mitigated cell reflux in vitro mimic, improved cell viability, reduced cell aggregation, and had an inhibitory effect on hypoxic reactions due to cell deposition with hiPSC-RPE. Additionally, gelatin hydrolysate did not affect cell adhesion and morphology, and decreased the expression of major histocompatibility complex class II molecules, which suggests reduced immunogenicity of hiPSC-RPE.

**Conclusion:**

Gelatin hydrolysate is considered a valuable and useful candidate for future regenerative therapies in hiPSC-RPE suspension transplantation.

## Highlights

• Gelatin hydrolysate reduces iPS-RPE cell reflux, enhancing viability and adhesion.
• Gelatin treatment reduces hypoxia cell death, promotes even distribution.
• It inhibits hypoxic reactions and decreases immunogenicity.
• A valuable candidate for regenerative therapies in RPE suspension transplantation.

## 1 Introduction

The retinal pigment epithelium (RPE), a melanin-containing cellular layer situated between the retinal and choroidal structures, plays pivotal roles in important processes the eyes, including in light absorption, conveyance of nourishment to the retina, phagocytosis of aged photoreceptor fragments, and maintenance of the blood-retina barrier [1,2]. Malfunction within the RPE can precipitate ocular impairments and disorders, such as age-related macular degeneration (AMD) and retinal dystrophy. Over the past decade, utilization of RPE derived from embryonic stem cells (ESC) or induced pluripotent stem cells (iPSC) has attracted significant attention as a promising cell-based regenerative therapy for such retinal degenerative conditions [[3], [4], [5], [6]] [[3], [4], [5], [6]] [[3], [4], [5], [6]]. Clinical studies in the field of retinal degeneration have pursued two main approaches: use of cell suspensions or utilization of an RPE sheet. Both methods have their own strengths and limitations. The cell suspension strategy for retinal regenerative therapy has advantages in terms of cellular accessibility and minimal invasiveness. However, it poses challenges in terms of precise targeting of the cells to the designated location and the potential risk of leakage of the cells into the vitreous humor, resulting in the formation of an epiretinal membrane (ERM) [7]. Addition of the Rho-associated coiled-coil forming kinase (ROCK) inhibitor Y-27632 promoted RPE cell adhesion, but did not contribute to the kinetics of the suspension itself for preventing reflux [8].

Gelatin, a denatured form of collagen, finds ubiquitous utility across diverse medical applications, including in regenerative therapy and pharmaceutical conveyance, due to its tenacious attributes, biodegradability, compatibility with biological systems, and easy clearance from the organism [9]. Previous studies have demonstrated the beneficial effects of gelatin microparticles in various applications, including its effects of improving the survival rate of cardiac stacked cell sheets, inducing capillary formation in microvascular networks, and inducing differentiation of stem cells towards muscle lineages [10,11]. Moreover, gelatin also serves as an efficacious vector for drug encapsulation among protracted-release drug delivery modalities [12]. Challenges exist in subretinal transplantation of conventional gelatin, characterized by a molecular mass of roughly 100,000, due to its notable viscosity and incompatibility with standard needles [13]. In this study, we examined the usefulness of gelatin hydrolysate, a soluble variant with a molecular mass of around 5000 through the formulation of a novel molecular weight distribution. Recent commercial availability of gelatin hydrolysate, coupled with its heightened fluidic attributes, positions it as a potentially useful reagent in suspension-based transplantation endeavors.

This study was conducted to assess the applicability of gelatin hydrolysate in Human iPS-RPE (hiPS-RPE) cell suspension transplantation, with particular emphasis on its potential to mitigate retrograde fluid dynamics subsequent to transplantation, as well as its suitability for enhancing the performance of RPE cells.

## 2 Methods

### 2.1 Gelatin hydrolysate preparation

The gelatin samples used were of porcine origin, and consisted of pig skin gelatin processed by alkaline extraction methods. Specifically, the samples belonged to the beMatrix™ gelatin series from Nitta Gelatin, Inc. (Osaka, Japan), the detailed characteristics of which have been reported by previous studies [14]. beMatrix™ gelatin was dissolved in PBS to prepare 1 %-20 % (w/v) gelatin hydrolysate, with the pH adjusted to 7.4 ± 0.1 with NaOH. The viscosity of the solution was measured with an MCR 302 rheometer manufactured by Anton Paar, Japan (cone plate R25, 1°, shear rate 200 s^−1^) at 25 °C. After the value was confirmed to be stable, the value at 1 min after the start of the measurement was adopted.

### 2.2 Preparation of hiPS-derived RPE cell suspensions

The hiPS-derived RPE (253G1) cells were differentiated using a previously described protocol [15]. hiPS-RPE cells were cultured in maintenance medium (DMEM/F12 [7:3] supplemented with B27 [Life Technologies, Carlsbad, CA] and 2 mML-glutamine [Sigma-Aldrich, St. Louis, MO]) supplemented with 10 ng/mL bFGF and 0.5 μM SB431542. The medium was changed every 2-3 days. The cells were cultured until near confluence, which took approximately 2-3 weeks. Upon reaching confluence, the cells were incubated with 0.25 % trypsin-EDTA (Life Technologies, Carlsbad, CA) at 37 °C in the presence of 5 % CO_2_ for 10 min. Trypsin was then neutralized with fetal bovine serum containing medium. The cell suspensions were prepared in Nunc tubes (Life Technologies, Carlsbad, CA) containing Opti-MEM medium (Thermo Fisher Scientific, Waltham, MA) at 5.0 × 10^6^ cells/mL. All experiments were performed using the cells from passage number 6-8.

### 2.3 Determination of the optimal concentration of the gelatin solution and measurement of refluxed cells

A retinal bleb model was created using polydimethylsiloxane (PDMS) soft lithography for quantification of the cellular reflux [16]. A 48-well plate was filled with PDMS, a cylindrical cavity with a diameter of 3 mm and height of 6 mm was made using a biopsy punch, and the cavity was covered with another thin PDMS sheet with a hole measuring 1 mm in diameter at the center (Supplemental Figure. 1ab). The cylinder space was filled with oxiglutatione intraocular irrigating solution, and 30 μL (1.5 × 10^5^ cells) of hiPS-RPE suspension was injected into it as a bleb mimic. After 30 min of static incubation, the lids were photographed to count the number of cells that had refluxed to the lid surface.

### 2.4 Cell viability assessment

The survival rates of hiPS-RPE suspensions stored in each of the suspension media (Opti-MEM, Opti-MEM containing 10 μM ROCK inhibitor, gelatin hydrolysate, and gelatin hydrolysate containing 10 μM ROCK inhibitor) were measured after 24 and 72 h of storage in an incubator at 37 °C in a 5 % CO_2_ atmosphere. Cell viability was assessed using trypan blue staining (0.4 %) (Life Technologies, Carlsbad, CA), in accordance with the manufacturer's instructions. The percentage of viable cells was calculated using a hemocytometer (WakenBtech Co., Kyoto, Japan) using a drop of the trypan blue/cell mixture. We then counted the trypan blue-negative cells (viable) and stained (dead) cells separately.

### 2.5 Cell adhesion test

The hiPS-RPE cell adhesion was evaluated by seeding the cells labeled with PKH fluorescent dyes (567 nm: Sigma-Aldrich: catalog no. PKH26GL) into 24-well plastic plates without any coating materials (1.0 × 10^6^ cells/well) in each of the following media: Opti-MEM, Opti-MEM containing 10 μM ROCK inhibitor, gelatin hydrolysate, and gelatin hydrolysate containing 10 μM ROCK inhibitor. The cells were incubated at 37 °C in a 5 % CO_2_ atmosphere for 24 h, washed 3 times with 500 μL of Opti-MEM medium, and fixed with 200 μL of 4 % paraformaldehyde (PFA). Cell images from 5 different locations (the center and positions above and below, and to the left and right of the center) in each well were captured by a camera attached to a fluorescence microscope (BZ9000, KEYENCE, Osaka, Japan). The average fluorescence intensity (arbitrary units) of these 5 images was calculated using the ImageJ software, and the average intensity was used as the result for each well.

### 2.6 Determination of the oxygen tension

To detect hypoxia in the hiPS-RPE cell suspensions, we used the hypoxia-detecting probe (mono azo rhodamine; MAR) (Goryo Chemical, Hokkaido, Japan), in accordance with the manufacturer instructions [17]. The hiPS-RPE cell suspension under each condition was incubated with 1 μM MAR containing 0.1 % dimethyl sulfoxide for 6 h. The fluorescence intensity was then analyzed by fluorescence microscopy (BZ9000, KEYENCE, Osaka, Japan). In order to determine the MAR fluorescence in individual cells, we used the Image J software to circle each cell boundary on phase contrast and superimposed the same image field under MAR conditions to measure the MAR fluorescence intensity within each cell boundary (Supplemental Fig. 2).

### 2.7 Evaluation of the effect of gelatin hydrolysate on the cell morphology

We examined the morphology of the hiPS-RPE by phase contrast microscopy and by immunostaining the Zona Occludens 1 (ZO-1). The hiPS-RPE cells were plated into a 24-well non-coated plate containing each of the media (Opti-MEM, Opti-MEM containing 10 μM ROCK inhibitor, gelatin hydrolysate, and gelatin hydrolysate containing 10 μM ROCK inhibitor) and cultured for 21 days at 37 °C in a 5 % CO_2_ atmosphere. No medium exchange was performed, and the maintenance medium was replenished every 3-4 days. The cell morphology was assessed by phase contrast and immunofluorescence microscopy (red, ZO-1). Scale bars = 50 μm.

### 2.8 Flow cytometry

The expression of HLA-class II molecules on the hiPS-RPE cells was evaluated. Before the assay, the hiPS-RPE cells were pretreated with recombinant human IFN-γ (100 ng/mL: R&D systems) for 48 h. In addition, before staining, the cells were incubated with a human Fc block antibody (Miltenyi Biotec, Auburn, CA, USA) at 4 °C for 15 min. After human Fc block staining, the RPE cells were stained with FITC-labeled anti-HLA-class II antibodies (HLA-DR, DQ, DP: BioLegend: catalog no. 361705) at 4 °C for 30 min. The RPE cells were also stained with FITC-labeled anti-mouse IgG at 4 °C for 30 min. The RPE samples were analyzed using fluorescence-activated cell sorting flow cytometer (FACSCanto™ II) (BD Biosciences).

To confirm the phagocytotic ability of the hiPS-RPE cells, hiPS-RPE cells were cultured with FITC-labeled porcine shed photoreceptor rod outer segments (ROS, 10 μg/cm^2^) at 37 °C in a 5 % CO_2_ atmosphere for 24 h. Control cells, that is, hiPS-RPE cells cultured in the absence of FITC-ROS, were also prepared [18].

### 2.9 Quantitative RT-PCR and ELISA

We examined the expressions of RPE-specific markers or inflammatory cytokines/chemokines in the RPE cells by quantitative RT-PCR and ELISA. Total RNA was isolated from hiPS-RPE cell lines (253G1) using the RNeasy Micro Kit (Qiagen, Venlo, Netherlands). After cDNA synthesis, the mRNA expressions of PEDF, bestrophin-1 (Best1), RPE65, transforming growth factor beta 2 (TGFβ2) and β-actin were determined by using qPCR Mastermix and highly specific Universal ProbeLibrary assays (all Roche Diagnostics, Mannheim, Germany) in triplicate, with LightCycler 480 (Roche Diagnostics, Basel, Switzerland). The tested primers and the Universal Probe for Best1, RPE65 and β-actin (control), as well as the PCR conditions, are described in Supplemental Table 1. Results indicate the relative expression levels of the molecules to β-actin (ΔΔCt: control cells = 1).

The concentrations of CCL2/MCP-1 (inflammatory cytokines/chemokines) in the supernatants of the hiPS-RPE cells in each medium were measured by using human CCL2/MCP-1 ELISA (R&D Systems, Minneapolis, MN, USA). VEGF and PEDF secretions by the hiPS-RPE cells in each medium were examined by using immunoassay kits for VEGF (VEGF Human ELISA Kit, Life Technologies, Carlsbad, CA) and PEDF (Human PEDF ELISA Kit, BioVendor, Brno, Czech Republic). The hiPS-RPE cells were seeded at 2.0 × 10^5^ cells/well into 24-well with non-coated plates and cultured at 37 °C in a 5 % CO_2_ atmosphere for 7 days. The medium was changed every 2 days, and fresh medium was added on the fifth day of incubation. After 48 h, the protein contents of the supernatants were examined.

### 2.10 Immunohistochemistry

The hiPS-RPE cells were fixed with 4 % paraformaldehyde for 20 min. Transplanted eyes that were collected at 14 days postoperatively were fixed in formaldehyde (Super Fix, Kurabo, Osaka, Japan) at 4 °C for one week and then embedded in paraffin (Sigma-Aldrich, St. Louis, MO). After paraffin block, the tissue was sliced into 10 μm. Samples were permeabilized with 0.2 % Triton X-100 in phosphate-buffered saline for 30 min, blocked with Blocking One (Nacalai Tesque, Kyoto, Japan) for 1 h at room temperature (RT), and incubated with primary antibodies diluted in Antibody Diluent (Dako, Glostrup, Denmark) overnight at 4 °C. The antibodies used are listed in Supplemental Table 2. Next, the cells were incubated with the secondary antibodies for 1 h at RT. The secondary antibodies were goat anti-rabbit Alexa Fluor 488 (Life Technologies, Carlsbad, CA) at 1:500 and goat anti-mouse Alexa Fluor 546 (Life Technologies, Carlsbad, CA) at 1:1000. Nuclei were stained with 4′,6-diamindino-2-phenylindole (DAPI) at 1:1000 (Life Technologies, Carlsbad, CA). Treatment with secondary antibodies was conducted for 1 h at RT, and images were obtained using a confocal microscope (LSM700; Zeiss, Jena, Germany and Leica-TCS SP8; Heidelberg, Germany) and fluorescence microscope (BZ-X800; KEYENCE, Osaka, Japan).

### 2.11 Subretinal injection of hiPS-RPE suspension in Japanese white rabbit eyes

All animal experimental protocols were approved by the Animal Care Committee of the RIKEN Center for Biosystems Dynamics Research and were conducted in accordance with Animal Research: Reporting of In Vivo Experiments (ARRIVE) guidelines.

To test the efficacy of gelatin hydrolysate on retaining the cells at the injected site, we subretinally injected hiPS-RPE suspension using Opti-MEM containing 10 μM ROCK inhibitor and a gelatin hydrolysate suspension containing 10 μM ROCK inhibitor, and compared the regurgitation and cellular persistence in the injected area. For postoperative observation, the hiPS-RPE cells were stained with PKH, and the suspension also contained 0.1 % of contrast dye (FLUORESCITE® Intravenous Injection). Japanese white rabbits (Kbl:JW) were purchased from KITAYAMA LABES Co., Ltd (Nagano, Japan). Rabbits were anesthetized with a mixture of ketamine and xylazine, and their pupils were dilated with 0.5 % tropicamide and 0.5 % phenylephrine hydrochloride. For transplantation of hiPS-RPE suspension, a complete vitrectomy (Constellation®, Alcon, Switzerland) was performed, and hiPS-RPE cells (2.0 × 10^5^ cells/50 μL) were injected into the subretinal space using PolyTip® cannula 25G/38G (MedOne Surgical, Inc., Sarasota, FL). The graft cells were monitored by color fundus photographs, autofluorescence (AF) (RetCamⅢ; Clarity, Pleasanton, CA and CX-1; Canon, Tokyo, Japan) and optical coherent tomography (OCT) (RS-3000; Nidek, Aichi, Japan) at 14 days after surgery.

## 3 Results

### 3.1 Determination of the optimal gelatin concentration

The objectives were first, to ascertain the potential of gelatin hydrolysate to mitigate the occurrence of reflux, and then, to ascertain the optimal gelatin concentration required for the suppression of reflux. To achieve this, we meticulously prepared a diverse array of gelatin samples containing gelatin of varying concentrations and molecular weights. Subsequently, we conducted a comprehensive assessment of the samples by evaluating the reflux phenomenon within the human SH-SY5Y neuroblastoma cells suspensions.

The numbers of leaked cells (percentage of leaked cells) for the samples suspended in Opti-MEM only, 1 %, 5 % and 20 % gelatin hydrolysate suspensions containing gelatin with a molecular weight of 5000, and 1 % and 5 % gelatin hydrolysate suspensions containing gelatin with a molecular weight of 20,000 were 9.93 ± 1.04 × 10^4^ (66.2 ± 6.9 %), 8.55 ± 0.29 × 10^4^ (57.0 ± 2.0 %), 3.45 ± 0.63 × 10^4^ (23.0 ± 4.2 %), 2.99 ± 0.55 × 10^4^ (19.9 ± 3.7 %), 6.78 ± 0.74 × 10^4^ (45.2 ± 4.9 %), and 3.85 ± 0.98 × 10^4^ (25.6 ± 6.5 %) (Fig. 1a). We then compared the numbers of refluxed cells for the suspensions containing gelatin hydrolysate with those for the suspensions containing a ROCK inhibitor. The number of leaked cells (percentage of leaked cells) for cell suspensions containing medium only, medium with a ROCK inhibitor, gelatin hydrolysate 5 % with a molecular weight of gelatin of 5000, and the same with a ROCK inhibitor added to it were 8.73 ± 2.31 × 10^4^ (58.2 ± 15.4 %), 9.55 ± 1.58 × 10^4^ (63.7 ± 10.5 %), 2.75 ± 0.89 × 10^4^ (18.3 ± 6.0 %), and 2.85 ± 1.25 × 10^4^ (19.0 ± 8.3 %). It was noteworthy that the count of refluxed cells was lowest for the hiPS-RPE cell suspension containing gelatin hydrolysate at a concentration of 5 % with or without a ROCK inhibitor (Fig. 1b).

**Fig. 1:**
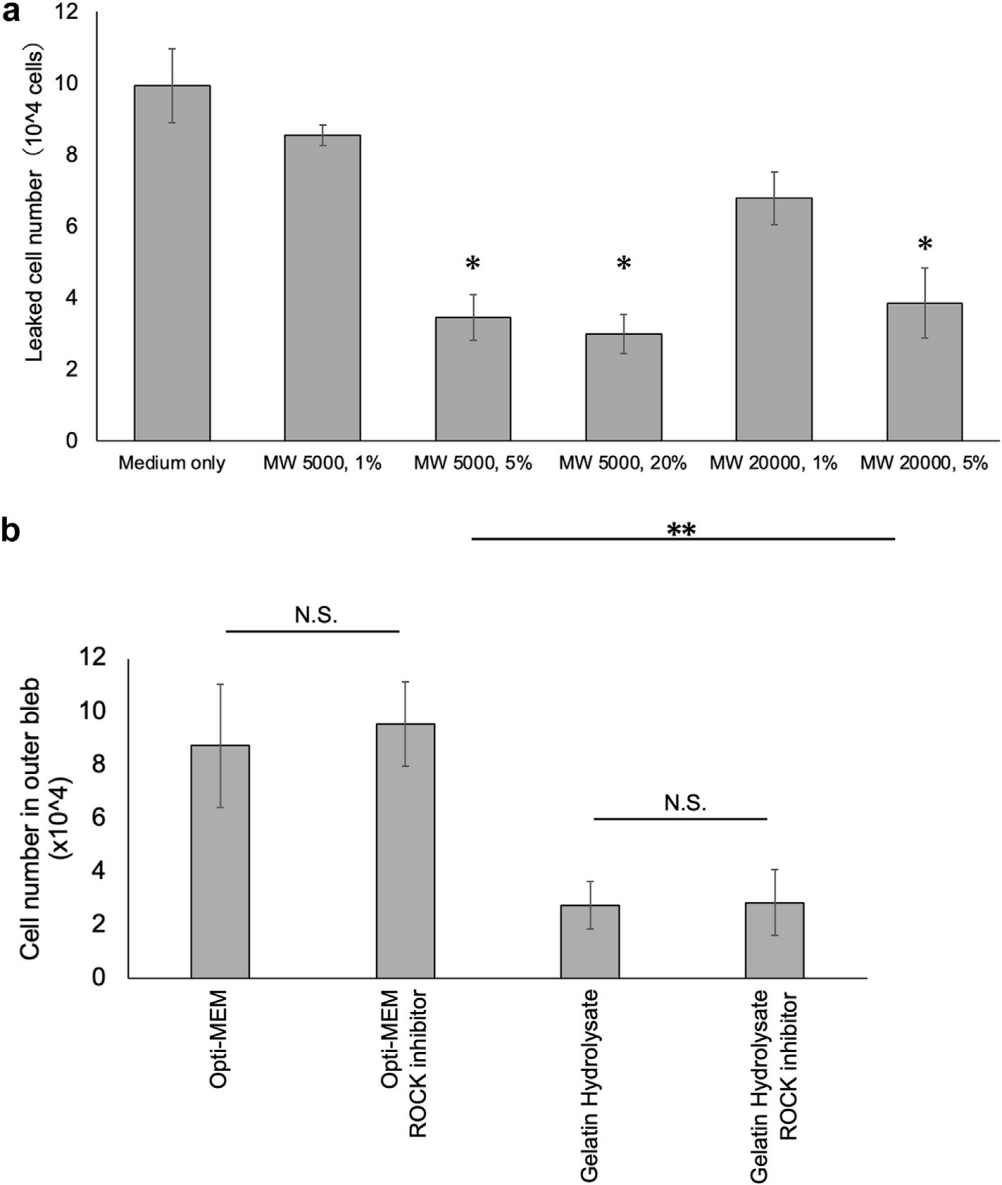
Evaluation of cellular reflux in a retinal bleb model. A retinal bleb model was filled with oxiglutatione intraocular irrigating solution, and 30 μL (1.5 × 10^5^ cells) of hiPS-RPE suspension was injected into the retinal bleb. After 30 min of static incubation, the number of leaked cells on top of the bleb lid was counted. (a) Number of leaked cells for each gelatin hydrolysate concentration and molecular weight used (n = 3; mean ± SD; ∗p < 0.05 compared to all other conditions; one-way ANOVA with Tukey's post hoc pairwise comparison). (b) Number of leaked cells with the use of gelatin hydrolysate and a ROCK inhibitor (n = 9; means ± SD; ∗∗p < 0.01 compared to all other conditions; one-way ANOVA with Tukey's post hoc pairwise comparisons).

### 3.2 Cell adhesion of hiPS-RPE cells suspended in gelatin hydrolysate containing or not containing a ROCK inhibitor

We investigated the cellular adhesion of hiPS-RPE cells suspended in gelatin hydrolysate containing or not containing a ROCK inhibitor by quantifying the PKH-positive cells on non-coated dishes after 3 washes following the static incubation for 24 h. As illustrated in Fig. 2a, most of the hiPS-RPE cells were washed out when the cells suspended in Opti-MEM medium alone were used. Conversely, the population of adherent cells increased in the presence of gelatin hydrolysate, which was further accentuated when the gelatin hydrolysate was supplemented with a ROCK inhibitor. The maximal number of adherent cells was, however, observed for cells suspended in Opti-MEM supplemented with a ROCK inhibitor, followed by those suspended in gelatin hydrolysate supplemented with a ROCK inhibitor, and then, cells suspended in gelatin hydrolysate alone (Fig. 2b). Thus, the adhesivity of the hiPS-RPE cells was found to increase with the addition of a ROCK inhibitors or gelatin hydrolysate to the cell suspension medium. It was also confirmed that the adhesion promoting effect of ROCK inhibitor was greater when it was added to Opti-MEM than to gelatin hydrolysate.

**Fig. 2:**
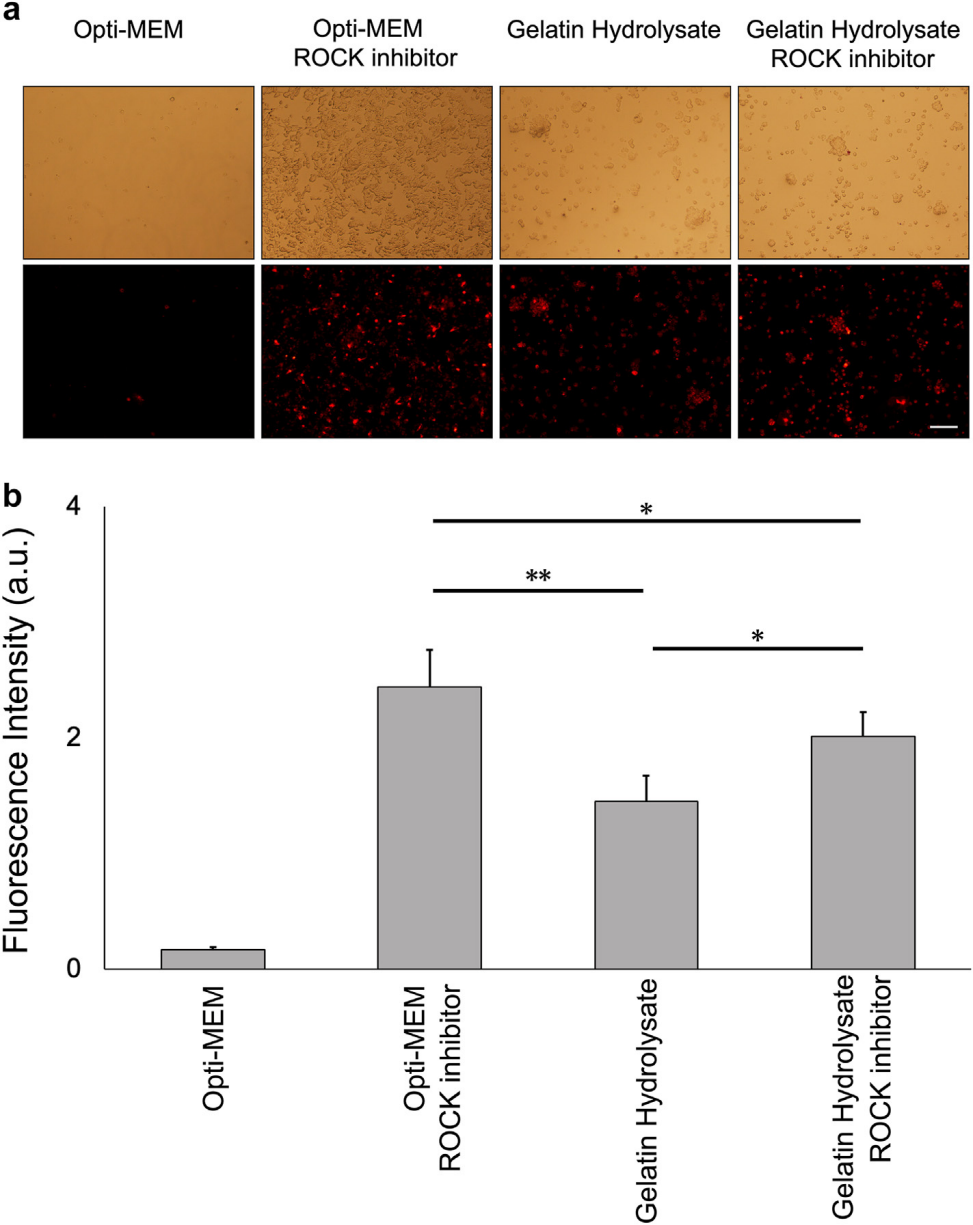
Effects of gelatin hydrolysate and ROCK inhibitor on the cell adhesion examined by comparing the following conditions; Opti-MEM, Opti-MEM containing 10 μM ROCK inhibitor, gelatin hydrolysate, and gelatin hydrolysate containing 10 μM ROCK inhibitor. The hiPS-RPE cells were labeled with PKH fluorescent dyes and seeded on to 24-well plates without any coating materials. The cells were incubated in the respective media at 37 °C in a 5 % CO_2_ atmosphere for 24 h, washed 3 times with 500 μL of medium, and fixed with 200 μL of 4 % PFA. (a) Representative cell images for each condition are shown: Opti-MEM, Opti-MEM containing 10 μM ROCK inhibitor, gelatin hydrolysate, and gelatin hydrolysate containing 10 μM ROCK inhibitor. (b) Quantitative analysis of the fluorescence intensity under each condition (n = 6; means ± SD; ∗p < 0.05 and ∗∗p < 0.01 compared to other conditions; one-way ANOVA with Tukey's post hoc pairwise comparisons).

### 3.3 Viability of the hiPS-RPE cell suspensions in each of the suspension media

Next, in order to estimate the viability of the RPE cells during and after injection, the impact of suspension of the cells in the different suspension media on the survival of the hiPS-RPE cells was investigated. The hiPS-RPE cells were suspended at 1.0 × 10^6^ cells/200 μL in Opti-MEM, Opti-MEM containing 10 μM of Y-27632, gelatin hydrolysate, and gelatin hydrolysate containing 10 μM Y-27632 and incubated at 37 °C in a 5 % CO_2_ atmosphere, for up to 72 h. Live and dead cells were counted using the standard trypan blue exclusion assay (Fig. 3a). After 24 h, the survival was about 42.8 ± 7.4 % in Opti-MEM and improved to 52.7 ± 11.8 % with the addition of a Y-27632 to Opti-MEM. In gelatin hydrolysate and gelatin hydrolysate containing Y-27632, the cell viabilities were 93.5 ± 5.7 % and 93.0 ± 9.6 %, with no significant difference between the two conditions. After 72 h, the viability was 12.4 ± 5.3 % in Opti-MEM and 25.7 ± 5.3 % in Opti-MEM containing Y-27632. The viability after 72 h in gelatin hydrolysate suspension improved to 81.3 ± 7.6 %, and that in gelatin hydrolysate containing Y-27632 improved to 84.1 ± 2.7 %, with no significant difference between the two conditions (Fig. 3b). These results indicate that, although the experimental setting cannot totally mimic the in vivo environment during and after transplantation, we could still presume that gelatin hydrolysate supplementation may support graft cell survival better as compared to ROCK inhibitor supplementation alone.

**Fig. 3:**
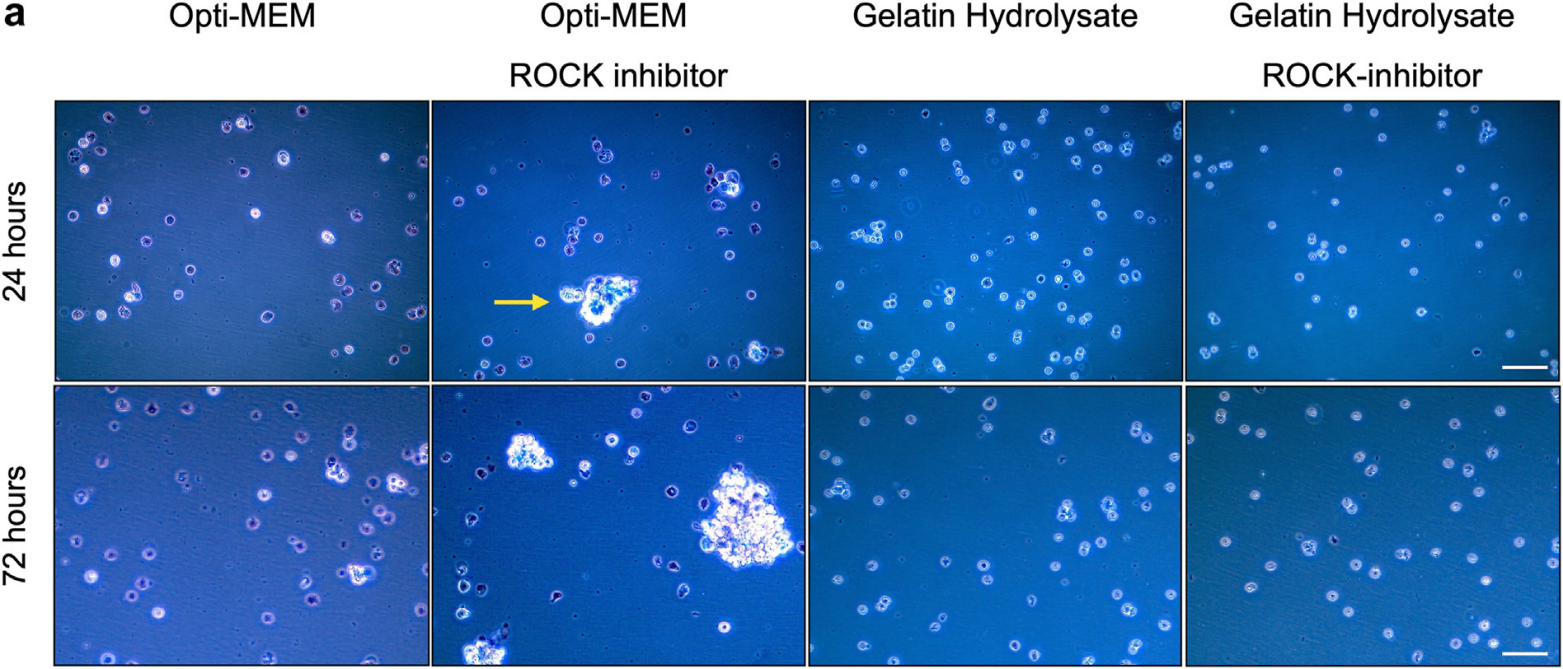

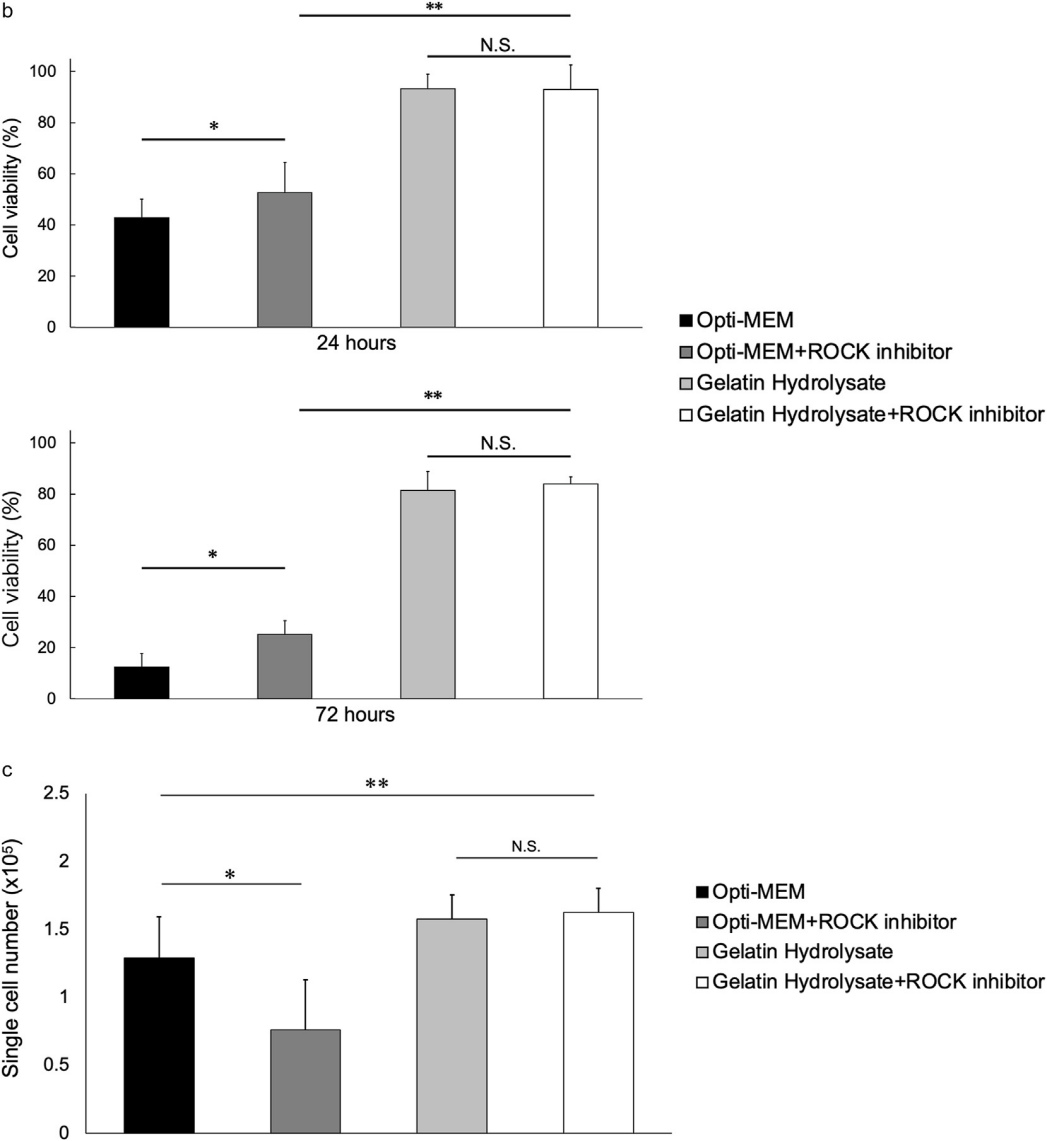
Time-course of preservation of the cell viability in hiPS-RPE cell suspensions under each condition; Opti-MEM, Opti-MEM containing 10 μM ROCK inhibitor, gelatin hydrolysate, and gelatin hydrolysate containing 10 μM ROCK inhibitor. The hiPS-RPE cell suspensions were stored for 24 and 72 h at 37 °C in a 5 % CO_2_ atmosphere. After 24 and 72 h of storage, the cell viability was assessed using trypan blue. (a) Trypan blue exclusion assay images of the samples under each condition. Cell aggregation is indicated by the yellow arrows. The photographs in the top row were after 24 h of storage, and those in the bottom row were after 72 h of storage. Scale bars = 50 μm (b) Quantitative analysis of viable cells under each condition described in Fig. 3a, after storage for 24 h and 72 h (n = 12; mean ± SD; ∗p < 0.05 and ∗∗p < 0.01 compared to all other conditions; one-way ANOVA with Tukey's post hoc pairwise comparisons). (c) Quantitative analysis of single cells under each condition described in Fig. 3a, after storage for 72 h (n = 12; means ± SD; ∗p < 0.05 and ∗∗p < 0.01 compared to all other condition; one-way ANOVA with Tukey's post hoc pairwise comparisons).

### 3.4 Gelatin inhibits the cell aggregation effect of ROCK inhibitors

We found that hiPS-RPE cells tend to aggregate in the presence of ROCK inhibitor in the cell suspension, consistent with a previous report (Fig. 3a) [19]. However, presence of gelatin hydrolysate in the cell suspension did not promote cell aggregation even in the presence also of Y-27632. We evaluated cell aggregation by counting individual isolated cells in 30 μL of the suspension medium (1.5 × 10^5^ cells) after 72 h of storage (Fig. 3c). The number of isolated cells in the medium-only condition was 1.29 ± 0.30 × 10^5^, whereas with the addition of Y-27632, the number became 7.63 ± 0.37 × 10^5^ cells, only about half the number as compared to that in medium alone. With the addition of gelatin hydrolysate to the suspension medium, the count was 1.58 ± 0.17 × 10^5^ cells, higher than that in the medium-only condition, and significantly higher, at 1.63 ± 0.18 × 10^5^ cells, with the addition of Y-27632 to the gelatin hydrolysate. This suggests that the suppression of cell aggregation by gelatin hydrolysate may result in the good survival of hiPS-RPE cells in a suspension medium containing gelatin hydrolysate.

### 3.5 Effect of the preservation condition on the oxygen tension

We previously reported that when hiPS-RPE cells are stored in suspension form, cell deposition occurs, resulting in hypoxia, which is associated with cell death [15]. In this study, we analyzed the effect of the composition of the suspension medium on the cells using the hypoxia detection marker, MAR (Fig. 4a). Cell aggregates show strong positivity for hypoxia markers, and the degree of positivity was the highest in the suspension medium containing a ROCK inhibitor condition, followed by that in the medium-only suspension. Significantly lower positivities were observed in the suspensions containing gelatin hydrolysate. Furthermore, there was no significant difference between suspensions containing gelatin hydrolysate alone and gelatin hydrolysate containing a ROCK inhibitor (Fig. 4b).

**Fig. 4:**
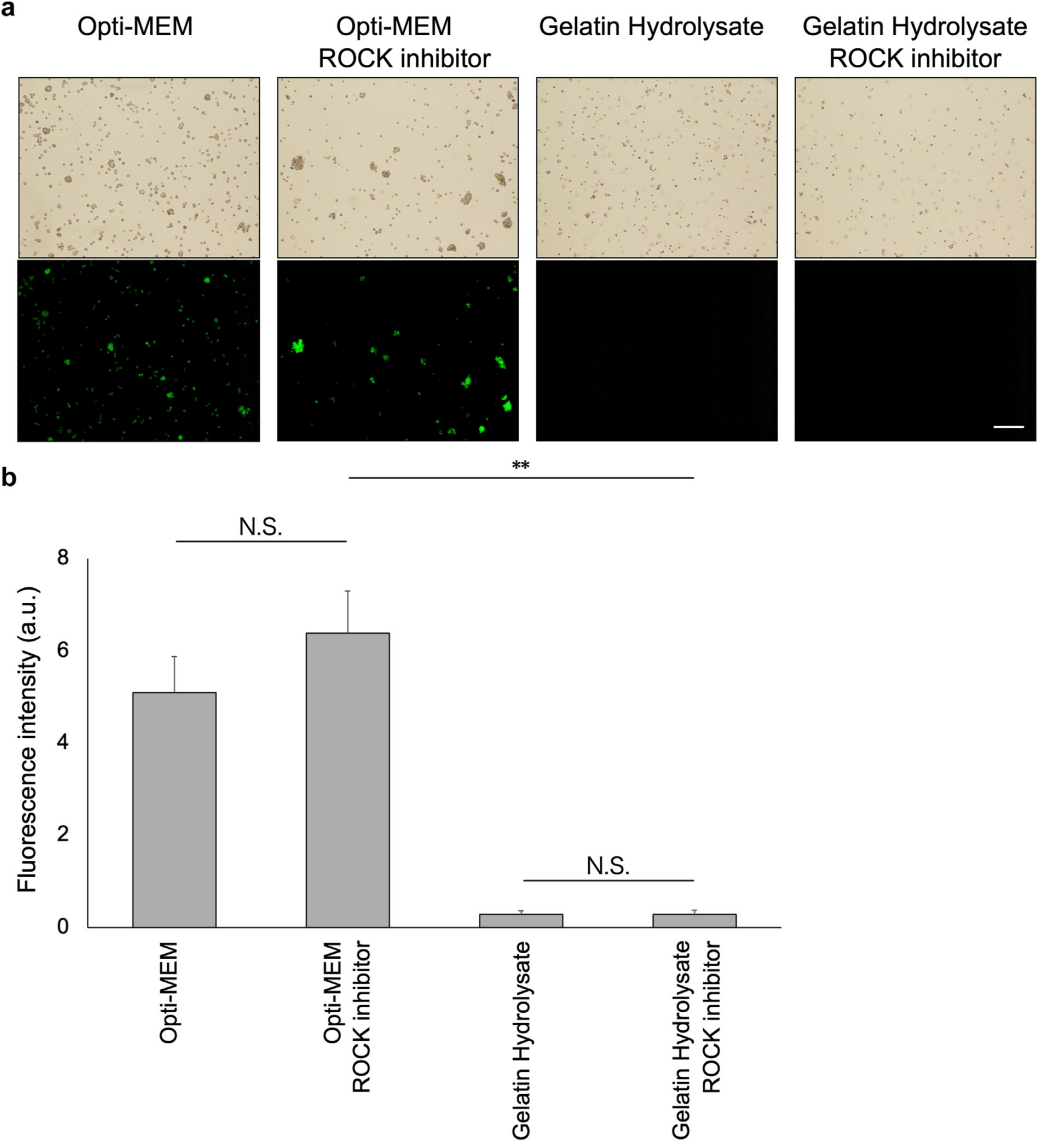
Hypoxia detection in the cells under the following conditions: Opti-MEM, Opti-MEM containing 10 μM ROCK inhibitor, gelatin hydrolysate, and gelatin hydrolysate containing 10 μM ROCK inhibitor. (a) Imaging of the cell hypoxia marker mono azo rhodamine (green, MAR) in hiPS-RPE cells under each condition after 6 h of incubation at 37 °C in a 5 % CO_2_ atmosphere. Scale bar = 50 μm (b) MAR fluorescence intensity in the same samples as those referred to in (a). (n = 6; means ± SD are presented. P values were calculated by one-way ANOVA with Tukey's post hoc pairwise comparisons (∗p < 0.05 compared to other conditions).

### 3.6 Evaluation of the effect of gelatin on the cell morphology

We confirmed the effect of gelatin hydrolysate on the cell morphology after cell settlement and growth. The hiPS-RPE cells were seeded in 24-well uncoated plates containing each of Opti-MEM, Opti-MEM containing 10 μM ROCK inhibitor, gelatin hydrolysate, and gelatin hydrolysate containing 10 μM ROCK inhibitor without coating agent and cultured for 3 weeks at 37 °C in a 5 % CO_2_ atmosphere, with the medium changed every 2 or 3 days. In the suspension containing medium alone, the cells were detached over time, and voids were observed here and there after 3 weeks. On the other hand, the cells were found to be firmly adherent for all other compositions of the suspension media, and the polygonal morphology of RPE cells was confirmed by ZO-1 immunostaining (Fig. 5).

**Fig. 5:**
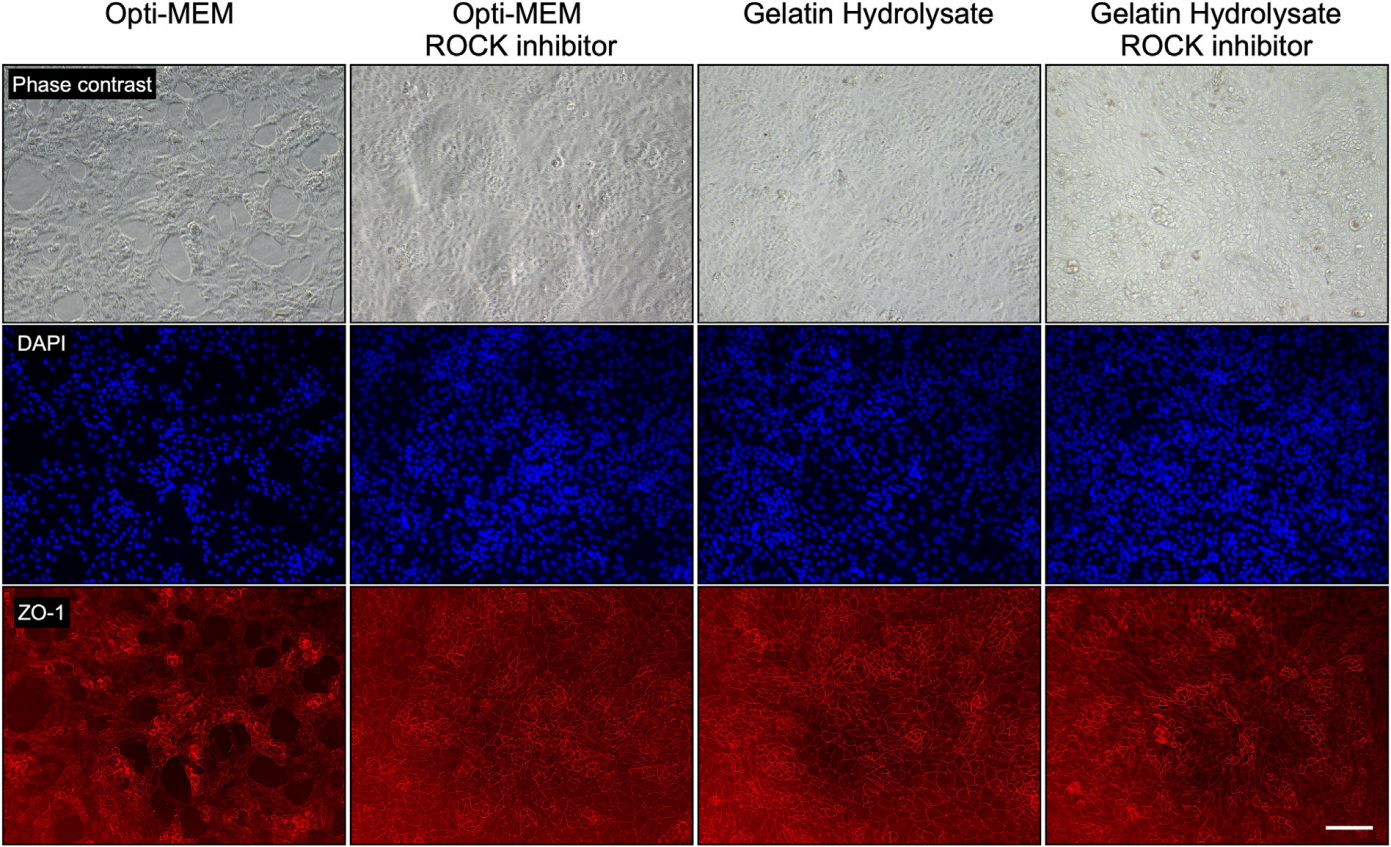
Epithelial morphology in culture of hiPS-RPE cells under each condition: Opti-MEM, Opti-MEM containing 10 μM ROCK inhibitor, gelatin hydrolysate, and gelatin hydrolysate containing 10 μM ROCK inhibitor. Cells were plated into a 24-well CELLstart-coated plate after preservation and cultured for 21 days. The cell morphology was assessed by phase contrast and immunofluorescence microscopy (red, ZO-1 and blue, DAPI). Scale bar = 50 μm.

### 3.7 Low immunogenicity of hiPS-RPE cells suspended in gelatin hydrolysate (HLA and ELISA)

Next, we examined the effects of cell suspension in gelatin hydrolysate on the induction of inflammation and immune responses elicited via major histocompatibility complex (MHC) class II molecule expression. MHC class II molecule-expressing RPE cells contribute to the immune and inflammatory responses elicited in the eye, by presenting superantigens to T lymphocytes [20]. We therefore examined the expressions of HLA-class II molecules (HLA-DR, DQ, and OP) on the hiPS-RPE cells in each of the suspension media. The RPE cells were pretreated with recombinant IFN-γ (100 ng/mL) for 48 h. Subsequent evaluation by flow cytometry revealed that as compared with the cells suspended in medium alone, those suspended in Y-27632 alone and in gelatin hydrolysate with/without Y-27632 showed significantly lower expression levels of HLA-class II molecules, as indicated by the mean fluorescence intensity (MFI) values (Fig. 6a and b).

**Fig. 6:**
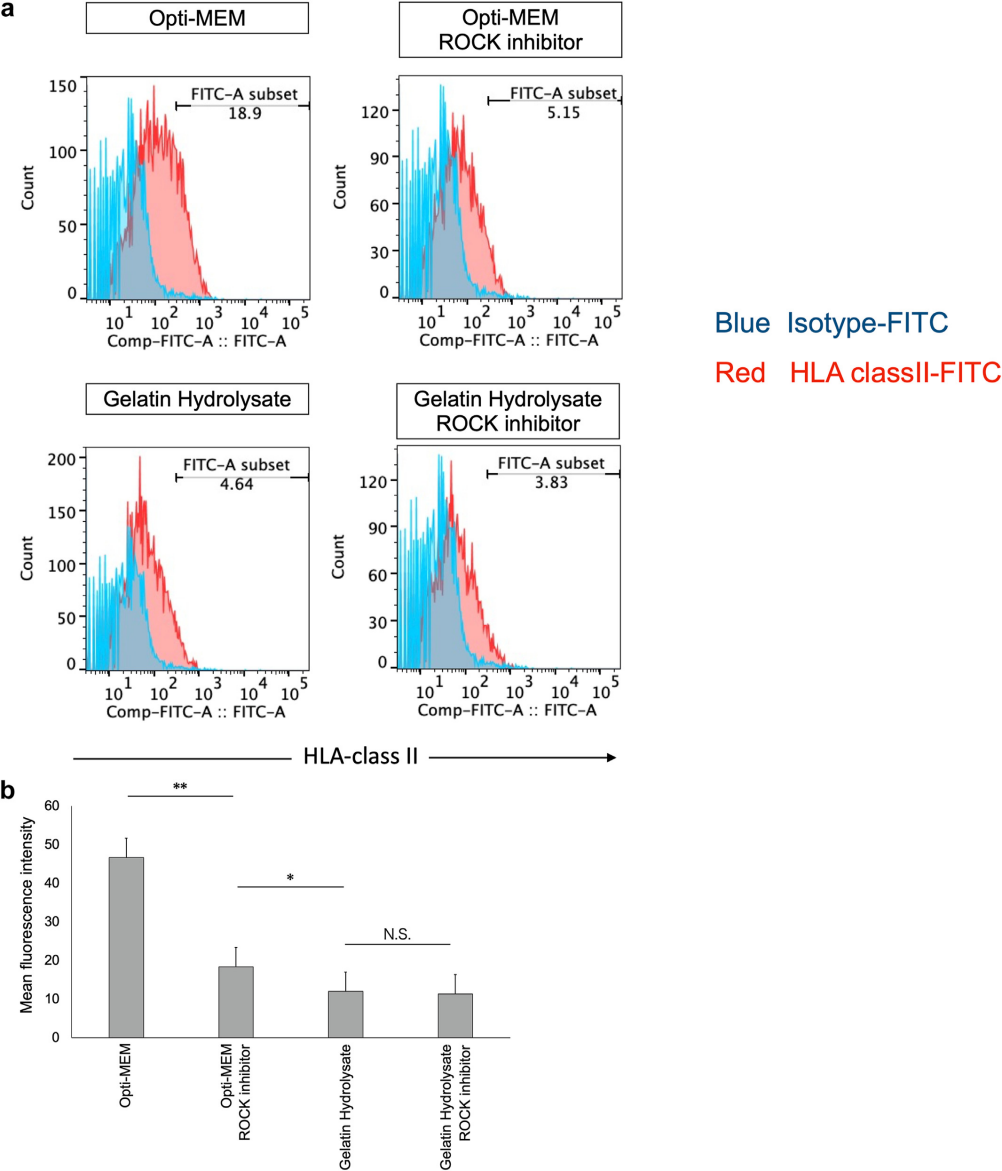
Expression of HLA-class II molecules on the hiPS-RPE cells under each condition: Opti-MEM, Opti-MEM containing 10 μM ROCK inhibitor, gelatin hydrolysate, and gelatin hydrolysate containing 10 μM ROCK inhibitor. hiPS-RPE cells were pretreated with recombinant human IFN-γfor 48 h. After human Fc block staining, the RPE cells were stained with FITC- labeled anti-HLA-class II antibodies at 4 °C for 30 min. The RPE cells were also stained with FITC-labeled anti-mouse IgG at 4 °C for 30 min. (a) Representative result of flow cytometry analysis. (b) Mean fluorescence intensity (MFI) of HLA class II molecule expression (n = 3; means ± SD; ∗p < 0.05 and ∗∗p < 0.01 compared to all other conditions; one-way ANOVA with Tukey's post hoc pairwise comparisons).

Next, we examined the induction of C–C motif chemokine ligand 2 (CCL2) and monocyte chemotactic protein 1 (MCP-1) as inflammatory cytokines/chemokines in the presence of gelatin hydrolysate by ELISA. ROCK inhibitor-treated hiPS-RPE cells were reported to show reduced expression of CCL2/MCP-1 by both qRT-PCR and ELISA as compared to the non-treated cells [8]. We obtained similar results for cells suspended in gelatin hydrolysate, with decreased secretion of CCL2/MCP-1 as determined by ELISA (Supplemental Fig. 3). These results indicate that gelatin hydrolysate suppresses inflammatory cytokines/chemokines, just like ROCK inhibitors.

### 3.8 Effect of gelatin hydrolysate on RPE cell function

In considering the clinical application of gelatin hydrolysate, we performed several quality control tests for hiPS-RPE to see if gelatin hydrolysate may affect the functioning of the cells. In the phagocytosis test, the hiPS-RPE cells were cultured in the presence of gelatin hydrolysate with shed photoreceptor rod outer segments (ROS) for 24 h and analyzed by flow cytometry. The results (Supplemental Fig. 4) revealed no apparent differences in the mean fluorescence intensity determined to evaluate the expression of ROS between cells suspended in medium alone, as control cells, and cells suspended under other conditions, indicating that the presence of gelatin hydrolysate had no significant effect on the phagocytotic function of the RPE cells. Second, cytokine/trophic factor secretion, one of the essential functions of RPE, was evaluated by ELISA, and the presence of gelatin hydrolysate again seemed to have no obvious effect on either VEGF or PEDF secretion (Supplemental Fig. 5). In addition, we examined the expression levels of RPE-specific markers (*PEDF, Best1, RPE65* and *TGFβ2*) by qRT-PCR; there seemed no decrease in the mRNA expression of any of these genes in the presence of gelatin hydrolysate as compared to the control medium group (Supplemental Fig. 6).

### 3.9 In vivo retinal observation after hiPS-RPE suspension injection

To test if the reflux is practically reduced in vivo by using gelatin hydrolysate, we injected fluorescein labeled hiPSC-RPE suspension into rabbits with or without gelatin hydrolysate (n = 3). Without gelatin hydrolysate, hyperfluorescence was observed in the whole vitreous space on postoperative day 1 by both fluorescence angiography (FA) mode and autofluorescence (AF) mode. Even at 14 days postoperatively, hyperfluorescence persisted in the whole vitreous space by AF mode. In contrast, with gelatin hydrolysate, hyperfluorescence was localized in both FA and AF modes on postoperative day 1. On postoperative day 14, hyperfluorescence was localized at the same area, suggesting the presence of injected cells at the injection site (Supplemental Fig. 7a and b). By OCT, with gelatin hydrolysate injection, high intensity reflection was observed under the retina, suggesting that injected cells subretinally remained, while without gelatin hydrolysate, a high reflection was observed on the retinal surface, possibly reflecting cellular reflux (Supplemental Fig. 7c). Following sacrifice, H&E staining of the sampled eyes revealed the presence of a single row hiPS-RPE like cells in the subretinal space (Supplemental Fig. 7d).

Next, we examined CD147 which was a transmembrane glycoprotein also known as EMMPRIN; extracellular matrix metalloproteinase inducer. In the gelatin hydrolysate, CD147-positive cells were present in a single row under the retina, whereas a few CD147-positive cells were found in Opti-MEM. Since the CD147 antibody used in this study was a human-specific antibody and used to identify human graft cells [21], the samples indicated that gelatin hydrolysate could retain iPS-RPE more efficiently under the retina compared to Opti-MEM (Supplemental Fig. 8a and b). In both the gelatin hydrolysate group and the Opti-MEM group, hiPS-RPE injection experiments were conducted on three rabbits each, revealing similar trends.

## 4 Discussion

While ROCK inhibitors have been acknowledged for their potential to promote iPS-RPE cell survival and cell adhesion [8], the postoperative complication of cell dispersion into the vitreous cavity remains unresolved yet with the addition of a ROCK inhibitor alone at the time of transplantation. Using a model designed to simulate subretinal transplantation, we demonstrated the ability of gelatin hydrolysate to markedly reduce the reflux of cells into the cavity and simultaneously improve the cell viability, representing an improvement over the effects of even a medium supplemented with ROCK inhibitors. As gelatin hydrolysate is somewhat viscous, it is better able to remain in the intended area of delivery during manipulation at the time of transplantation. It suppresses reflux of the cells into the cavity, promotes cell survival and adhesion, and then eventually undergoes degradation and absorption, thereby facilitating cell engraftment and function.

First, we analyzed the dose-dependence of the effect of viscosity in inhibiting cell reflux inhibition, which suggested that a relatively low molecular weight is the optimal factor for reflux inhibition. The underlying reasons to explain the effect of gelatin in reducing reflux due to gelatin could include an increase in the viscosity of injectable solutions containing gelatin and/or the formation of gelatin-based gels subsequent to exposure to an aqueous tissue environment, as suggested by previous studies [22,23]. Furthermore, gelatin with an elevated viscosity and high molecular weight might take a higher force and a longer time before dissolution following injection [24].

The inhibition of ROCK significantly contributes to the promotion of cell viability across various cell lineages. This includes the potent suppression of apoptosis in human ESC, often metaphorically referred to as the death dance, and suppression of apoptosis in cardiomyocytes and corneal cells [19,25] [[19], [25], [26], [27]] [[19], [25], [26], [27]]. Previous studies have shown that during storage of hiPS-RPE cell suspensions, the induction of hypoxia as a result of cellular deposition leads to cell death [15]. Results using MAR showed that the response to hypoxia was significantly decreased in hiPS-RPE cells subjected to gelatin hydrolysate treatment. The results of cell adhesion confirmed by PKH staining also showed that a ROCK inhibitor was more effective in medium alone than in gelatin hydrolysate. Taken together, these findings suggest that gelatin hydrolysate forms distinct physical intercellular spaces amidst RPE cells to reduce cellular aggregation and deposition. Moreover, during a 3-week culture, hiPS-RPE cells showed homogeneous cellular dispersion, indicating an even distribution of cells even during the seeding process.

In light of the immunogenic nature of RPE cells, strategies aimed at mitigating intraocular inflammation following transplantation need to be considered [28]. Previous reports have shown that ROCK inhibitors exert immunosuppressive effects, including suppressing allogeneic immune responses and graft-versus-host disease [[29], [30], [31]] [[29], [30], [31]] [[29], [30], [31]]. In this study, we found that gelatin hydrolysate showed comparable efficacy to that of ROCK inhibitors in suppressing MHC class II molecule expression. MHC class II molecules present peptide antigens to T cells and downregulated expression of these molecules generally implies weak immune responses [32]. As suggested by the results of our in vitro assays, downregulation of MHC class II molecules on hiPS-RPE cells through application of both a ROCK inhibitor and gelatin hydrolysate might be expected to reduce the risk of graft rejection. Also, gelatin hydrolysate did not seem to have much relation to the induction of inflammation. Yet, immune responses to gelatin hydrolysate remain to be further tested in vivo studies. More importantly, the impact of gelatin hydrolysate on the expression of distinct RPE markers or on the process of phagocytosis of the rod outer segments (ROS) appeared to be insignificant. This lends support to the rationale for the use of gelatin hydrolysate in RPE transplantation.

In the cell injection without gelatin hydrolysate, the fundus examination showed hyperfluorescence in the entire intraocular space as captured by FA mode from the first postoperative day, suggesting that reflux had occurred soon after injection. In addition, OCT and histological analysis showed that there was almost no hiPS-RPE remaining under the retina, which was consistent with the results observed in the vitro experiments. In contrast, with gelatin hydrolysate, localized hyperfluorescence was observed in the fundus photographs and hiPS-RPE cells were subretinally identified by OCT and histological analysis, which were positive for human-specific CD147. This may suggest that gelatin hydrolysate retained hiPS-RPE at injected space while gelatin gradually dissolved.

## 5 Conclusions

In conclusion, the findings of this study suggested that gelatin hydrolysate suspension may be suitable for use as a medium for hiPS-RPE cell suspensions intended for subretinal injection. The gelatin hydrolysate may also be useful as a preservation solution, as it was found to be effective for maintaining cell survival for a certain period of time. Thus, gelatin hydrolysate can be used continuously from the time of cell production to the time of injection, and appears to be a versatile and convenient material to use.

## Author contributions

S.K. conceived the study. S.K. and H.I. performed the examinations. S.S. designed and performed the immune experiments. S.K., T.S., and K.K. performed the cell injection experiment. S.K. and M.M. analyzed the image data. S.K. statistically analyzed the data. S.K. wrote the manuscript, which was approved by all authors prior to submission. M.M., Y.T., S.S., K.K., and M.T. provided helpful guidance and suggestions.

## Funding information

Supported by a Bayer Retina Award and Grant-in-Aid for Early-Career Scientists (21K16902).

## Declaration of competing interest

The authors declare no conflict of interest.

## Figures and Tables

**figs1:**
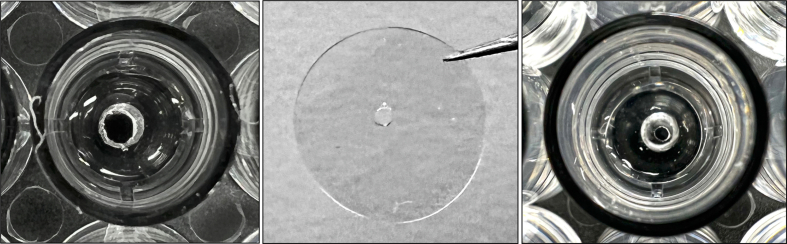


**figs2:**
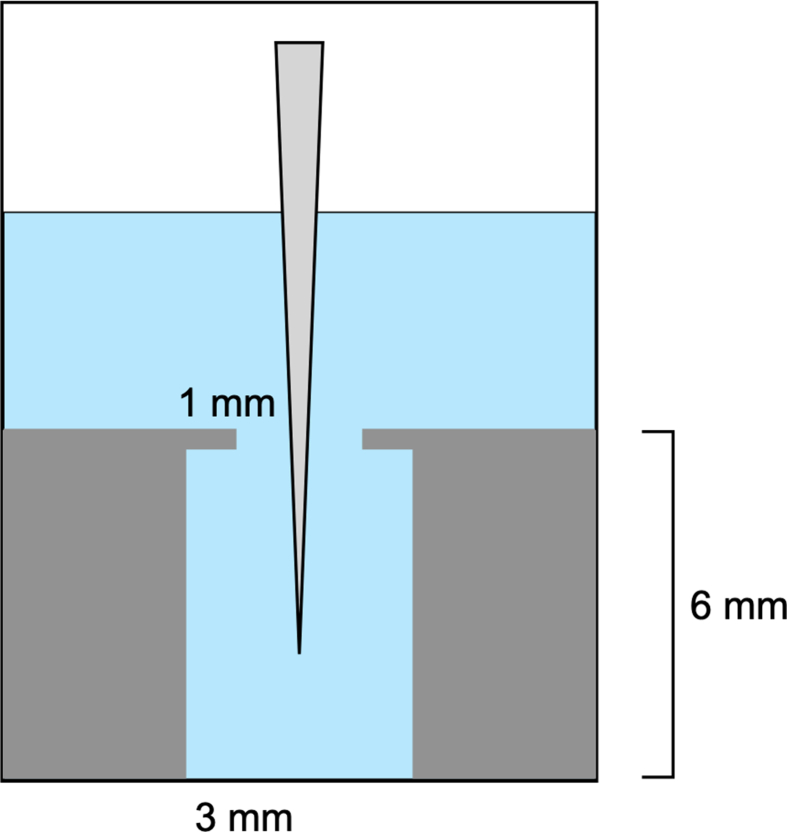


**figs3:**
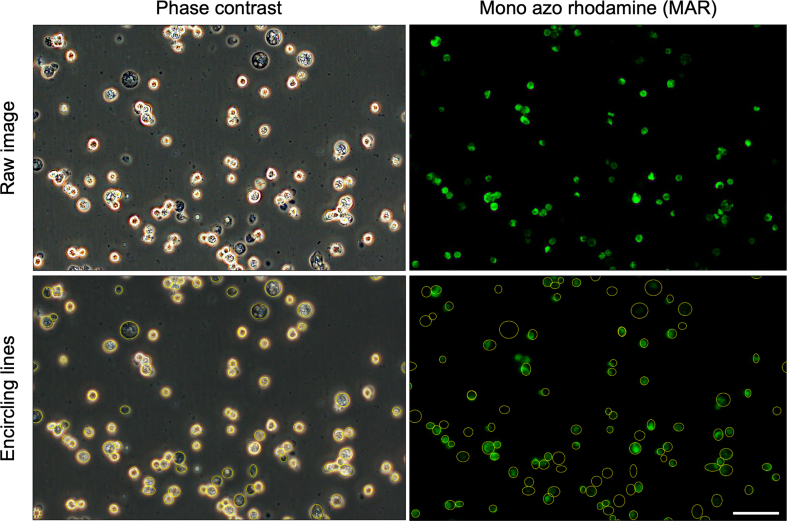


**figs4:**
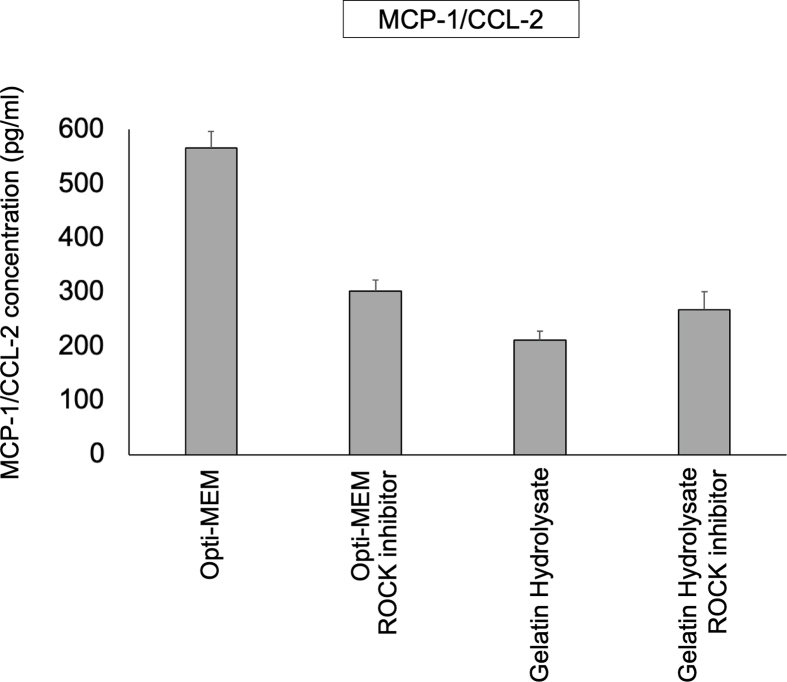


**figs5:**
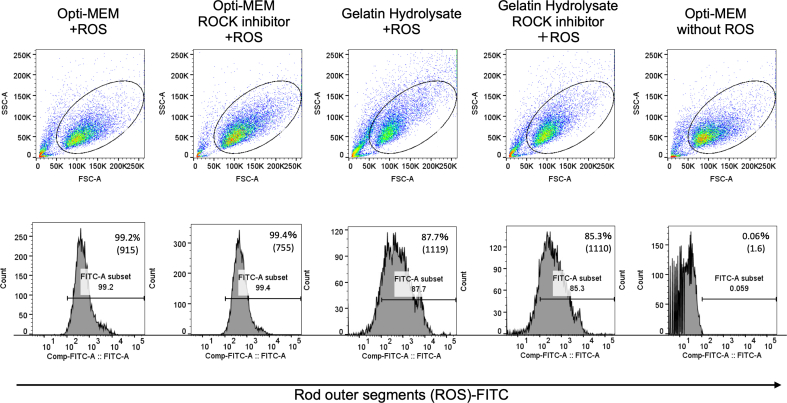


**figs6:**
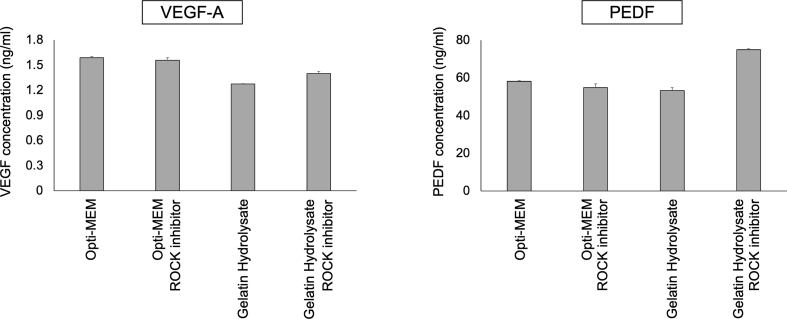


**figs7:**
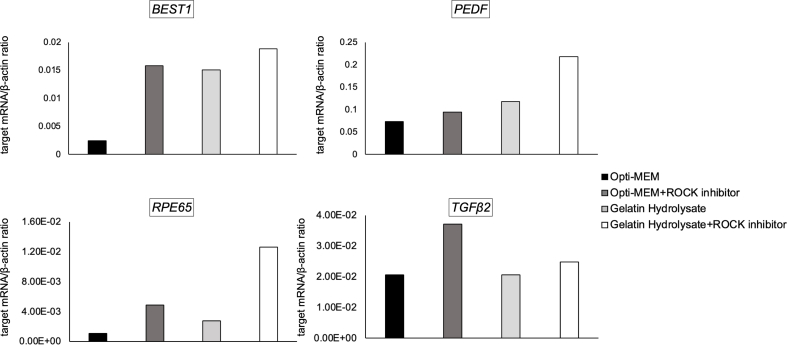


**figs8:**
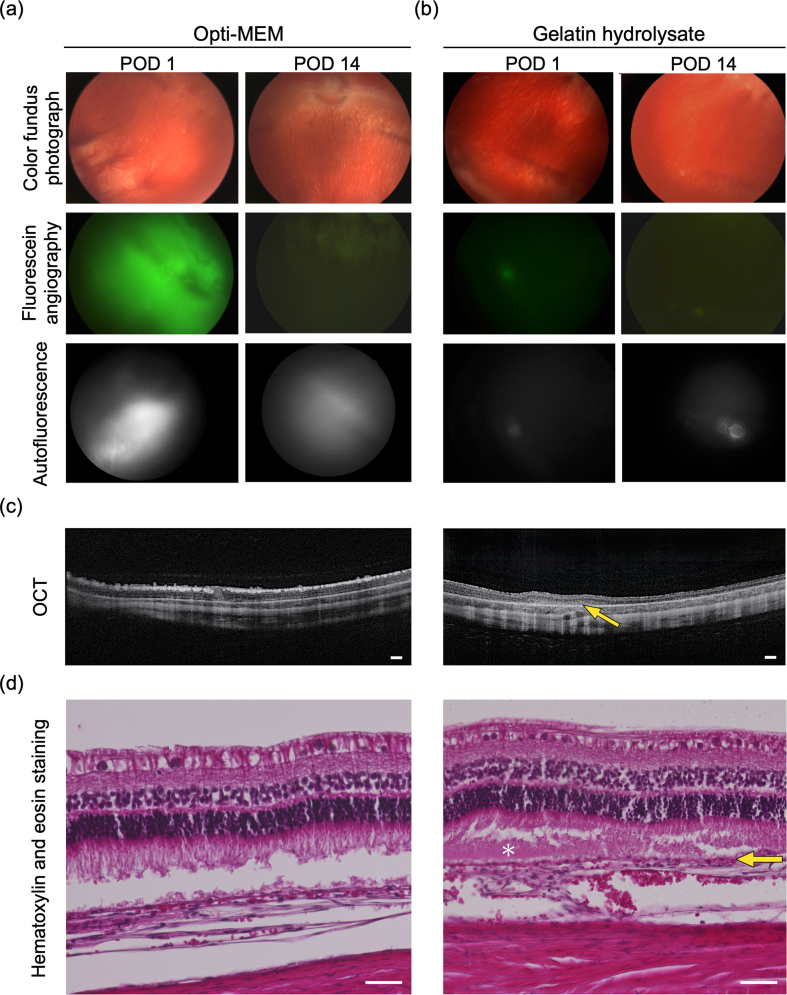


**figs9:**
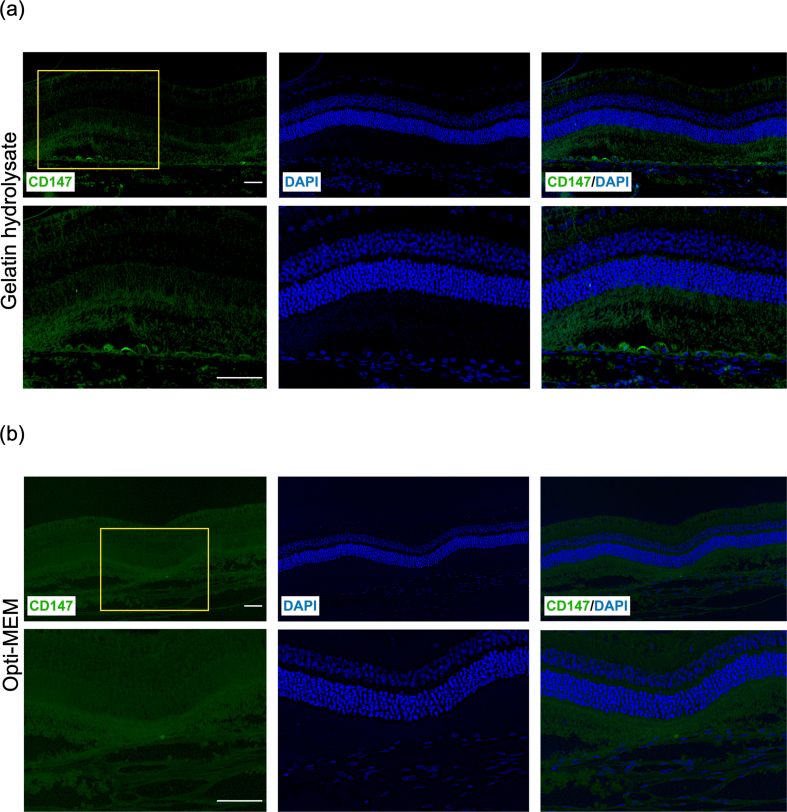


**figs10:**
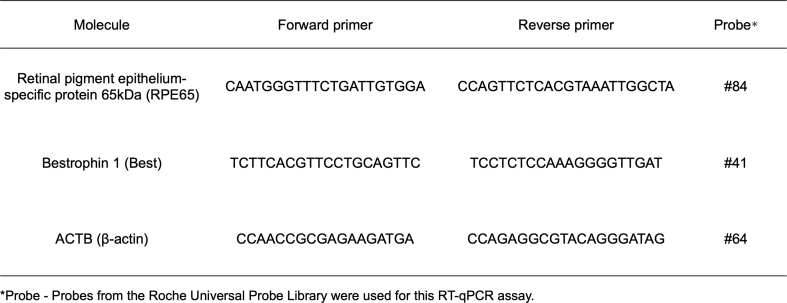


**figs11:**